# Possible protective effect of natural flavanone naringenin-reduced graphene oxide nanosheets on nonalcoholic fatty liver disease

**DOI:** 10.1007/s00210-024-03495-9

**Published:** 2024-10-16

**Authors:** Doaa Abdelmoneim, Ehab B. Eldomany, Mohamed El-Adl, Ahmed Farghali, Gehad El-Sayed, El Said El-Sherbini

**Affiliations:** 1 Biochemistry and Chemistry of Nutrition Department, Faculty of Veterinary Medicine, Mansoura University, Mansoura, 35516 Egypt; 2 Biotechnology and Life Sciences Department, Faculty of Postgraduate Studies for Advanced Sciences, Beni-Suef University, Beni-Suef, 62511 Egypt; 3 Material Science and Nanotechnology Department, Faculty of Postgraduate Studies for Advanced Sciences, Beni-Suef University, Beni-Suef, 62511 Egypt

**Keywords:** High caloric, Fast food, NAFLD, Naringenin, Reduced graphene oxide

## Abstract

Utilizing naringenin as a safe, natural compound for reducing graphene oxide and to determine whether Nar-RGO more effectively mitigates the harmful effects of HFFD-induced NAFLD compared to crude naringenin. Using a straightforward experimental setup, we utilize the bioactive flavonoid naringenin (NAR) as the reducing agent to synthesize naringenin-reduced graphene oxide nanosheets (Nar-RGO). Naringenin loading on graphene oxide was validated using electroscopic methods (SEM and TEM) and zeta potential measurements. Utilization of reduced graphene oxide for naringenin encapsulation resulted in a significant improvement in hepatic steatosis, insulin resistance, oxidative stress, and signs of inflammation in HFFD-induced NAFLD compared to crude naringenin. This study demonstrates that Nar-RGO exhibits significantly greater efficacy compared to free naringenin. Therefore, it can be used as a promising medicine in counteracting high-fat-fructose diet (HFFD)-induced NAFLD.

## Introduction

Approximately 25% of the global adult population is affected by nonalcoholic fatty liver disease (NAFLD), a potentially severe liver disease that has substantial adverse effects on health and carries social and economic implications (Xu et al. 2022). The NAFLD spectrum encompasses different abnormalities, ranging from steatosis to nonalcoholic steatohepatitis (NASH), which involves various degrees of necrotic inflammation, fibrosis, and, ultimately, cirrhosis (Pafili and Roden 2021). NAFLD is a significant factor in the development of hepatocellular carcinoma (HCC), which is the fourth leading cause of cancer-related mortality worldwide (J. D. Yang et al. 2019). Liver transplantation is a compelling alternative. However, its effectiveness is hindered by organ rejection and a scarcity of donors (Hernández-Aquino and Muriel 2018). Furthermore, NAFLD has been associated with a higher risk of cardiovascular disorders and type-2 diabetes mellitus (T2DM)-related complications such as neuropathy and nephropathy (Zaharia et al. 2019). This implies that a holistic approach is required for the treatment of NAFLD.

NAFLD is usually asymptomatic, so diagnosis usually follows the incidental finding of abnormal liver enzymes and lipid profile (Dyson et al. 2014). Abundant studies and clinical practices have proven that the liver enzyme levels such as ALT and AST were increased usually in NAFLD patients, and these liver enzymes could be used as the diagnostic markers for NAFLD (Martin-Rodriguez et al. 2017). Moreover, 20–80% of NAFLD patients also have dyslipidemia, which is characterized by high triacylglycerol (TAG) and low high-density lipoprotein cholesterol (HDL-C) levels (Targher et al. 2010). Additionally, low albumin levels can indicate liver dysfunction and are commonly associated with advanced stages of NAFLD, such as fibrosis or cirrhosis. Additionally, albumin is a marker for overall nutritional status and inflammation, both of which are relevant in NAFLD cases​ (Qi et al. 2024). Recent studies highlight that interleukin levels, particularly IL-6, are often elevated in NASH patients compared to those with milder forms of NAFLD. Measuring interleukins helps in the early detection of liver inflammation, offering a non-invasive method to predict disease progression (Beiriger et al. 2023). Furthermore, monitoring adiponectin levels can help assess the degree of liver inflammation and fibrosis, making it a potential biomarker for disease severity and progression in NAFLD patients (Gatselis et al. 2014).

Various factors have been proposed as contributors to the development of NAFLD, including insulin resistance (IR), oxidative stress, mitochondrial dysfunction, genetic, epigenetic, and environmental factors, along with poor lifestyle (such as the consumption of energy-rich food and the lower physical activity) (Noureddin and Sanyal 2018). Specifically, reports have indicated that consuming excessive quantities of fructose leads to a higher incidence and prevalence of NAFLD (Roeb and Weiskirchen 2021). Fructose metabolism is unregulated in the liver because it bypasses the rate-limiting process of glycolysis, making it a significant factor in the production of lipids (Ajah et al. 2022). Therefore, immediate intervention is required to address metabolic risk factors and raise global awareness in order to decrease the future burden of NAFLD-related disorders (Huang et al. 2021). This is particularly significant given that NAFLD is reversible through prompt diagnosis and treatment (Sherif 2019).

Several drugs are currently being studied for their potential to prevent or protect against NAFLD by targeting hepatic inflammation, TAG accumulation, oxidative stress, or liver fibrosis (Rotman and Sanyal 2017). However, the potential protective role of flavonoids has received limited interest. Flavonoids, naturally occurring polyphenol compounds, have been found to regulate lipid metabolism, inflammation, insulin resistance, and oxidative stress. In addition, they have several therapeutic applications (Tan et al. 2022). Given the growing association between chemically manufactured drugs and adverse side effects, it is worth considering plant flavonoids as a potential new anti-NAFLD medication in the future.

The 5000 known flavonoid compounds can be categorized into at least seven subgroups based on variation in structure. These subgroups include flavones, isoflavones, flavonols, flavanols, flavanones, anthocyanidins, and chalcones. Three rings, specifically C6-C3-C6, represent the essential structure of flavonoids. The bioactivity of these compounds is determined by the patterns of structural substitution in their C6-C3-C6 rings (Shen et al. 2022). Naringenin, which is the aglycone form of naringin, belongs to the subclass of flavanones. It is primarily found in citrus fruits, such as orange, lemon, mandarin, and grapefruit (Manchope et al. 2017). Naringenin demonstrates several pharmacological properties, such as hepatoprotective, nephroprotective, anti-diabetic, anti-inflammatory, anti-hypolipidemic, antioxidant, and anti-cancer activities (Bhia et al. 2021). Studies have shown that naringenin is a bioactive compound that is generally safe and non-toxic. However, its low water solubility limits its bioavailability. In addition, its low bioavailability is attributed to factors such as extensive gastrointestinal disintegration, liver metabolism, restricted membrane transportation, rapid metabolism, and elimination from the human body (Arafah et al. 2020).

These obstacles can only be solved by implementing new tools and technology. Effective diagnosis and treatment are becoming more feasible by developing advanced nanosystems with controlled size, shape, and surface functions (Gnanasekar et al. 2020). One of these methods is the use of encapsulation techniques (Gomes et al. 2023). Current research is focused on two-dimensional (2D) nanomaterials, which are garnering significant interest due to their distinct physicochemical characteristics with small atomic thicknesses. A variety of two-dimensional (2D) materials have been developed for energy, environmental, and medical applications. These materials include graphene, black phosphorus, transition metal dialchonoginides (TMDs), layered double hydroxides (LDHs), transition metal oxides (TMOs), and graphite-like carbon nitrides (gC3N4) (Mohammadpour and Majidzadeh-A 2020). Graphene oxide has attracted considerable attention due to its strength, extensive surface area, relatively low weight, chemical stability, improved cell adhesion, differentiation, and application in tissue repair (Bellet et al. 2021). Furthermore, graphene-based biosensors demonstrate high sensitivity, facilitating the early detection and monitoring of a range of diseases, including cancer and neurological disorders (Badillo-Ramírez et al. 2022). The antimicrobial properties of graphene offer the potential to address antibiotic-resistant pathogens, representing a promising approach to the global healthcare challenge posed by infectious diseases (Kanchanapally et al. 2015). The widespread application of graphene-based materials in various studies, coupled with the increasing interest in their industrial use at large scales, has raised significant concerns regarding the potential toxicity of graphene in biological systems (Kiew et al. 2016). Previous studies have indicated that pristine graphene and GO, when not subjected to additional surface modification following inhalation and intravenous injection, can lead to significant pulmonary inflammation and granuloma formation. Conversely, surface functionalized GO tend to exhibit significantly lower toxicity (K. Yang et al. 2013). Another in vivo study demonstrated that prolonged exposure to GO resulted in opacity in the animals’ eyes, whereas reduced graphene oxide (RGO) showed biosafety (An et al. 2018). A study by Wang et al. (2011) suggests that RGO exhibits a certain level of biocompatibility, as evidenced by both in vitro and in vivo experiments showing that RGO can be safe, contingent upon (Liao et al. 2018; Wang et al. 2011).

Reduction methods focus on eliminating oxygen functional groups present in GO to reinstate the sp^2^ carbon network of graphene, leading to RGO that exhibits improved chemical stability, high substance loading efficiency, good dispersion, and easy functionalization. The synthesis methods for obtaining RGO raise concerns regarding toxicity, particularly those involving hydrazine, hydroquinone, and sodium borohydride (Agarwal and Zetterlund 2021). Concerns regarding environmental issues have prompted numerous researchers to develop reduced graphene oxide through a process termed green reduction of graphene oxide. Various approaches include the utilization of natural reducing agents, such as plant extracts (polyphenols) (Dos Santos et al. 2024). However, a significant challenge associated with this green route pertains to its efficiency relative to the synthesis route utilizing hydrazine, as well as obtaining RGO with good properties.

Therefore, this research aims to use naringenin as a safe, natural compound to reduce graphene oxide and to determine whether the combination of naringenin and RGO (Nar-RGO) mitigates the harmful effects associated with HFFD-induced NAFLD more effectively than crude naringenin.

## Material and methods

### Animals

A total of 32 male Sprague–Dawley rats (140–180 g) were purchased from the Animal House, Faculty of Science, Zagazig University. The animals were housed for a period of 7 days prior to the start of the experiment. The experiment was carried out in an animal house facility at the Faculty of Veterinary Medicine, Mansoura University, Mansoura, Egypt. The rats were housed in four cages per cage on a 12-h light/dark. Additionally, rats had free access to water and standard rodent chow. The animal protocol was designed to minimize pain or discomfort to the animals according to the research ethical committee guidelines in the Faculty of Veterinary Medicine, Mansoura University, Mansoura, Egypt. The study was conducted following the guidelines of the Basic & Clinical Pharmacology & Toxicology policy for experimental and clinical studies (Tveden-Nyborg et al. 2023).

### Chemicals and reagents

Naringenin was purchased from Sigma-Aldrich, Germany (product number: BCCF4512). Graphene oxide (two to five layers with a diameter of 7.5 µm) was purchased from Nanografi, Turkey (product number: NG01GO0102). Fructose was purchased from Alpha Chemika, India. TRizol reagent was purchased from Geneaid, Taiwan (product number: MI00505). For the determination of liver enzymes, levels of glucose, TAG, total cholesterol, lipid peroxides, superoxide dismutase (SOD), and glutathione peroxidase (GPx), colorimetric kits were obtained from BioDiagnostic Company (Egypt). The sandwich ELISA method was used for adiponectin and insulin concentration. The kit was purchased from SinoGeneClon Biotech Co., China, and Bioactive Diagnostics, respectively. For serum proinflammatory markers, enzyme-linked immunosorbent assay (ELISA) was used.

### Synthesis of Nar-RGO

Nar-RGO was synthesized according to Shanmuganathan et al. (2020) with minimal modifications. In brief, 60 mg of graphene oxide (GO) was mixed with 80 ml of distilled water under constant stirring for 20 min. Subsequently, a solution containing 50 mg of naringenin in 20 ml of ethanol was added dropwise to the mixture, followed by the addition of 50 µl of ammonium hydroxide solution dropwise. The mixture was maintained for 1 h, continuously stirring at 90°֯C. After reduction completion, the Nar-RGO was repeatedly centrifuged at 13,000 rpm for 20 min and collected. Finally, the pellet was re-suspended with Milli-Q water and lyophilized to obtain a purified Nar-RGO powder.

### Characterization of Nar-RGO

The characterization of Nar-RGO nanoparticles was achieved via multiple investigative techniques.

#### Scanning electron microscopy (SEM)

The surface morphology was characterised using the scanning electron microscopy** (**SEM) technique. Before conducting the SEM analysis, the samples were placed on aluminum stubs and then sputtered with a gold layer for 2 min to avoid electrical charging during examination. SEM images were taken at the EM unit at the PSAS faculty at BSU. Images were obtained using a field emission scanning EM (Zeiss Sigma 500 VP Analytical FE-SEM, Carl Zeiss, Germany).

#### Transmission electron microscopy (TEM)

The fine morphological nature of synthesized samples was investigated using a transmission electron microscope (TEM, JEOL-JEM-2100 with a 200 kV accelerating voltage). Samples for TEM analysis were prepared by drying a droplet of material suspension on a carbon-coated copper grid.

#### Zeta-potential measurements

The surface charge and the stability of the Nar-RGO were assessed by measuring the zeta potential. The measurements were performed by dynamic light scattering using a Zetasizer Nano-ZS System (Malvern).

#### Encapsulation efficiency (EE)

The encapsulation efficiency (EE) of the Nar-RGO was determined as previously described (Zhang et al. 2023). The nanoparticles were freeze dried, re-dissolved in water, and then diluted with 70% ethanol before being vortexed. To separate free naringenin from the nanoparticles, the solution was centrifuged at 14,000 rpm at 4 °C for 45 min. Naringenin content was quantified using a UV spectrophotometer, measuring absorbance at a wavelength of 288 nm. The encapsulation efficiency was calculated using the following equation:

$$\text{EE }(\text{\%}) = \frac{(\text{total naringenin amount}-\text{free naringenin amount})}{\text{total naringenin amount}} \times 100$$

### Study design

The rats were weighed and then randomized into four groups (*n* = 8). The rats were subjected to daily treatment for a duration of 8 weeks as follows: Group 1: fed with a standard chow diet. Group 2 (HFFD): given 60% fructose orally and fed on a high-fat diet, as shown in Table 1. Group 3: given HFFD and received 50 mg/kg naringenin according to Liu et al. (2022) and Rashmi et al. (2018) dissolved in 0.5% carboxy methyl cellulose (CMC) solution by oral gavage. Group 4: given HFFD with Nar-RGO orally. In order to eliminate potential bias, all groups were administered an equal amount of CMC in which naringenin was dissolved.

**Table 1:** Ingredients of standard diet and high-fat diet

Ingredients	Standard diet	High-fat diet
Protein (soya bean meal)	28%	23%
Carbohydrates (yellow corn)	57%	50%
Fat	10% (corn oil)	22% (palm oil)
Other ingredients	5%	5%

Per 1 kg vitamin–mineral premix contains 12,000 IU vitamin A, 2400 IU vitamin D3, 20 mg vitamin E, 4 mg vitamin K3, 3 mg vitamin B1, 7 mg vitamin B2, 25 mg niacin (vit. B3), 10 mg pantothenic acid (vit. B5), 5 mg vitamin B6, 15 μg vitamin B12, 50 μg biotin, 1 mg folic acid, 50 mg vitamin C, 100 mg Mn, 60 mg Fe, 60 mg Zn, 5 mg Cu, 2 mg I, 500 μg Co, 150 μg Se

At the conclusion of the experiment (8 weeks), chow was removed from cages 12 h prior to capitation. Rats were anesthetized by intraperitoneal injection of Na thiopental at a dose of 50 mg/kg.B.wt. dissolved in 5 ml sterile distilled water.

### Preparation of serum

The blood samples were collected and divided into two portions. The first portion was collected in a Na fluoride tube for glucose concentration determination. The second portion was allowed to clot, and serum was separated following centrifugation for 20 min at 3000 rpm. Then, clear supernatant was transferred to clean, dry Eppendorf tubes and stored at – 20 °C until the measurement of insulin concentration (Chevenne et al. 1998), total cholesterol concentration (TC) (Allain et al. 1974), and high-density lipoprotein cholesterol (HDL-C) (Lopes-Virella et al. 1977). Alanine transaminase (ALT), aspartate transaminase (AST) activities, albumin concentration, adiponectin concentration, and IL-β and IL-6 concentrations were also measured. Insulin resistance was assessed by measuring HOMA-IR according to Matthews et al. (1985).

### Liver homogenate preparation

Livers were dissected from the rats, rinsed in normal saline, and then homogenized in PBS (PH 7.6). Homogenates were centrifuged at 4000 r.p.m. for 20 min. Afterward, the clear supernatant was removed by micropipette and stored at – 80 °C until the measurement of hepatic malonaldehyde (MDA) concentration (Draper and Hadley 1990), hepatic superoxide dismutase (SOD) activity (Nishikimi et al. 1972), and hepatic glutathione peroxidase (GPx) activity (Paglia and Valentine 1967).

### Histopathological examination

The liver tissues were fixed using 10% neutral buffered formalin for hematoxylin and eosin staining (H&E). Other liver tissues were immediately frozen and subjected to oil-red staining.

### Quantitative real-time polymerase chain reaction

Using Trizol, total RNA was extracted from hepatic tissues, followed by cDNA synthesis using a cDNA synthesis kit. Quantitative real-time polymerase chain reaction (qRT-PCR) was conducted using the SYBR Green Kit Cat. No. 204141 to target the PPAR α, sterol regulatory element-binding protein (SREBP)-1c, and fatty acid synthase (FAS) genes. Table 2 shows primer sequences for target genes. All mRNA levels were normalized using the GAPDH level.

**Table 2:** Primers used for quantitative real-time polymerase chain reaction

Gene	Forward primer	Reverse primer	NCBI GenBank
GAPDH	CAACGGGAAACCCATCACCA	ACGCCAGTAGACTCCACGACAT	NM_017008.4
PPAR α	GCATGGCTGAGAAGACGCTTG	GGATAGCCTTGGCAAATTCCG	NM_013196.1
SREBP-1c	ACAGCACAGCAACCAGAAACTC	TTCATGCCCTCCATAGACACAT	NM_001276708.1
FAS	TTGGCTTAGTGATTGCATCTCGT	CAGGGTCTCTGTCCTCCTTTTGT	NM_139194.2

### Statistical analysis

The statistical analysis was determined using SPSS 22.0 software. Data were expressed as means ± standard error of the mean (SEM). Using one-way ANOVA, we compared means by utilizing the tabulated *F* test to determine the significance value. We also utilized Tukey’s and post hoc tests to determine significant differences between means. The level of statistical significance was set at a *p*-value of 0.05.

## Results

### Characterization of Nar-RGO

#### Scanning electron microscopy (SEM)

**Fig. 1:**
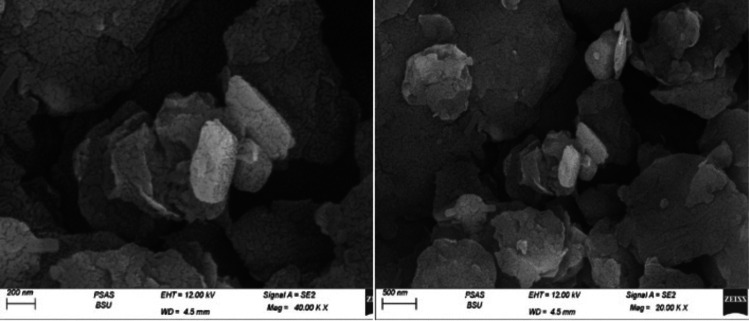
Morphological characterization and structure images of graphene oxide using SEM showed the fine layers and thickness

**Fig. 2:**
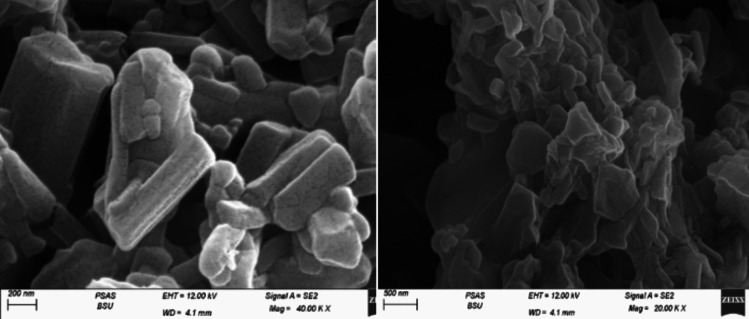
Morphological characterization and structure images of naringenin powder using SEM showed fine layers of the powder

**Fig. 3:**
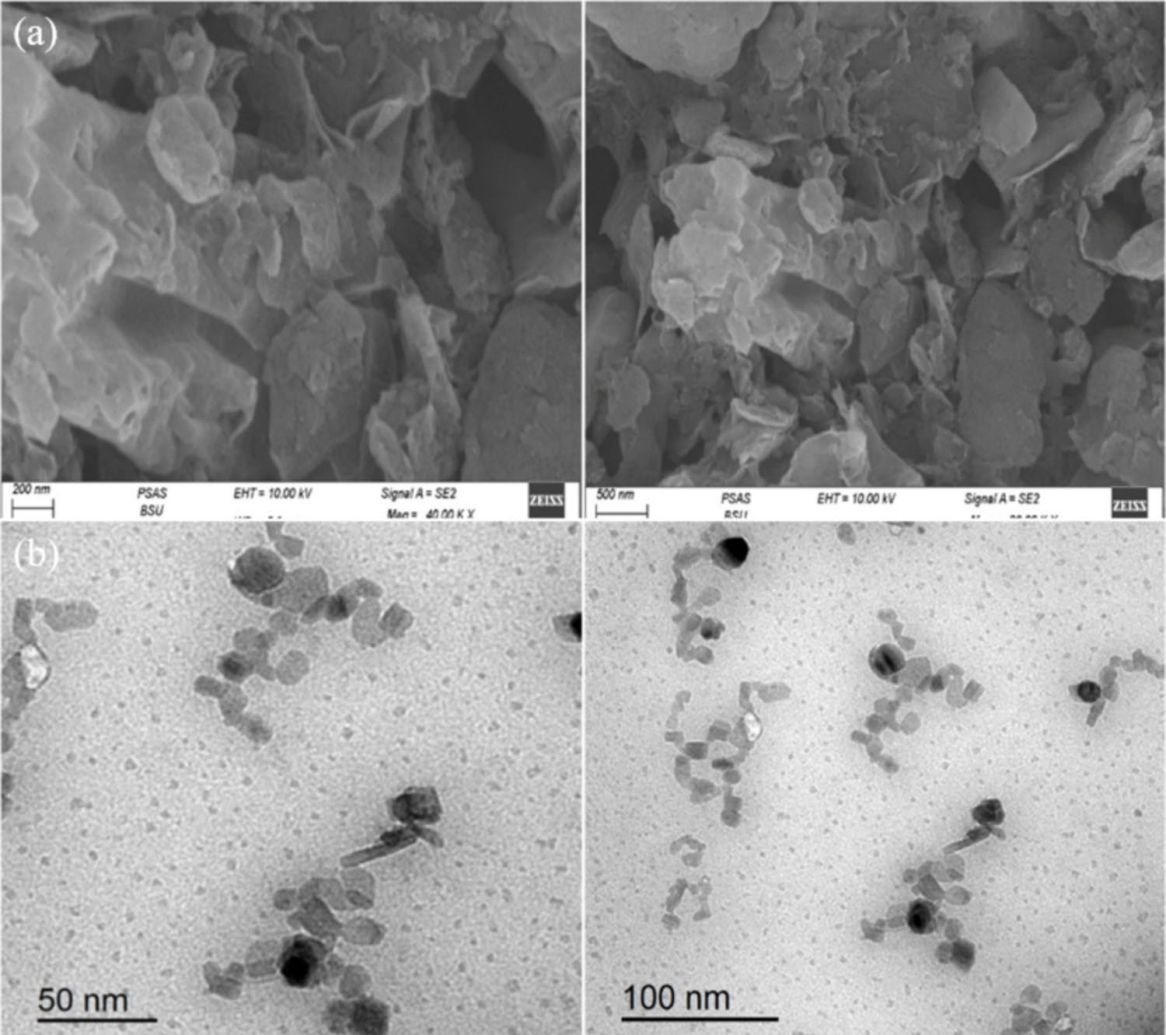
Images of prepared naringenin with graphene oxide using SEM (upper panels) and TEM (lower panels) showed the loading of naringenin on graphene oxide layers

The scanning electron microscopy (SEM) analysis of graphene oxide reveals the existence of flakes, which are smaller in size, more clumped, and stacked as depicted in Fig. 1. Additionally, the SEM analysis of naringenin, as shown in Fig. 2, reveals fine layers of the powder. On the other hand, the SEM images of Nar-RGO reveal well-separated platelets of RGO that are closely associated with each other, with naringenin well embedded on the surface of the RGO sheets (Fig. 3a). This embedding of naringenin suggests a strong interaction between the naringenin molecules and the RGO matrix.

#### Transmission electron microscopy (TEM)

In Fig. 3b, highly magnified TEM micrographs of Nar-RGO reveal uniformly distributed and well-dispersed spherical naringenin molecules on the surface of the transparent, silky, and wavy reduced graphene oxide nanosheets. The observed morphology in the TEM micrographs supports the findings from the SEM images, highlighting the effective incorporation of naringenin within the RGO structure.

#### Zeta potential

**Fig. 4:**
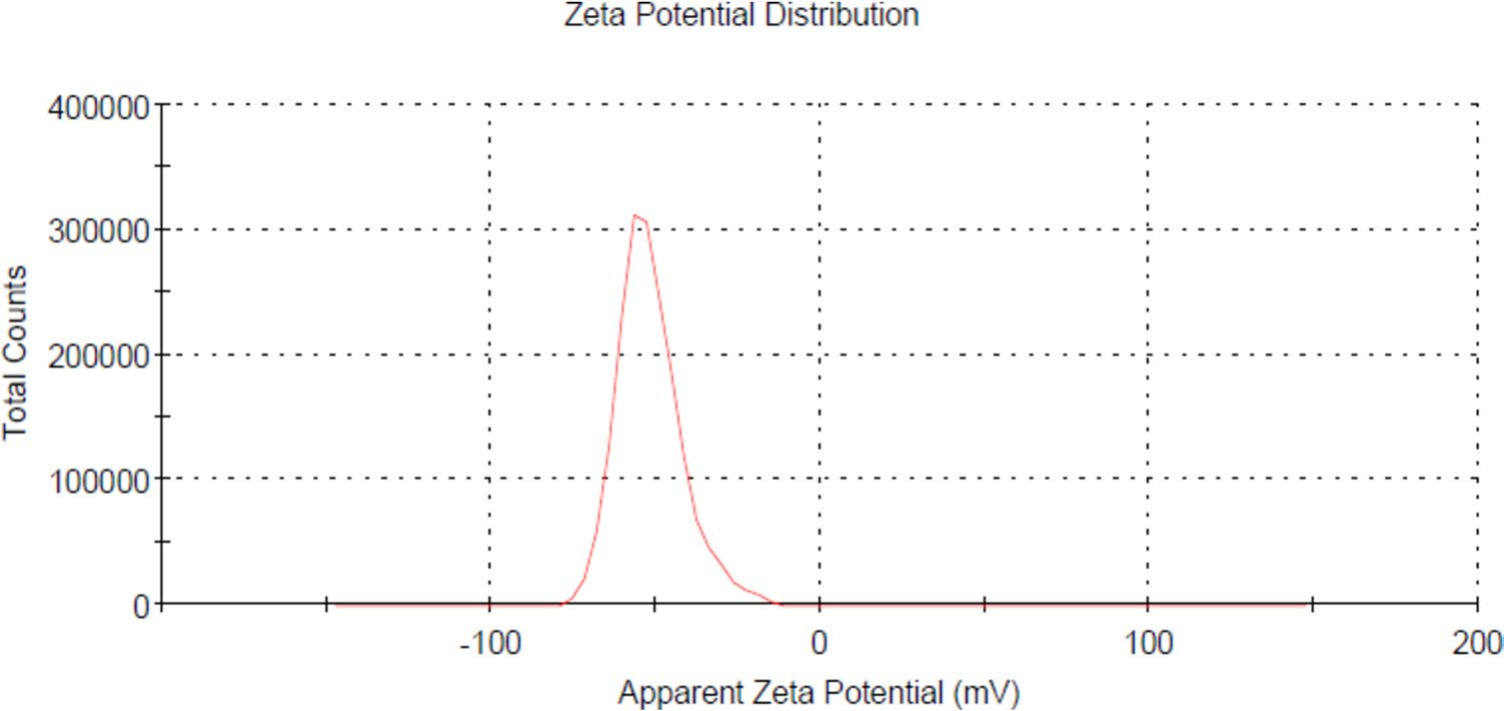
Zeta potential measurement of Nar-RGO

The data revealed that the average zeta potential was − 50.8 mV (Fig. 4), indicating that manufactured Nar-RGO has a significant anionic activity contributing to their colloidal stability in suspension. The negative zeta potential suggests that these nanoparticles have a strong repulsive force, which prevents aggregation and helps maintain a stable dispersion​.

#### Encapsulation efficiency (EE)

The encapsulation efficiency was 68%, meaning that 68% (34 mg) of the total naringenin used in the formulation was successfully encapsulated, while the remaining 32% (16 mg) was either free or lost during the process.

### Effect of naringenin and Nar-RGO on serum damage markers in rats exposed to HFFD

**Fig. 5:**
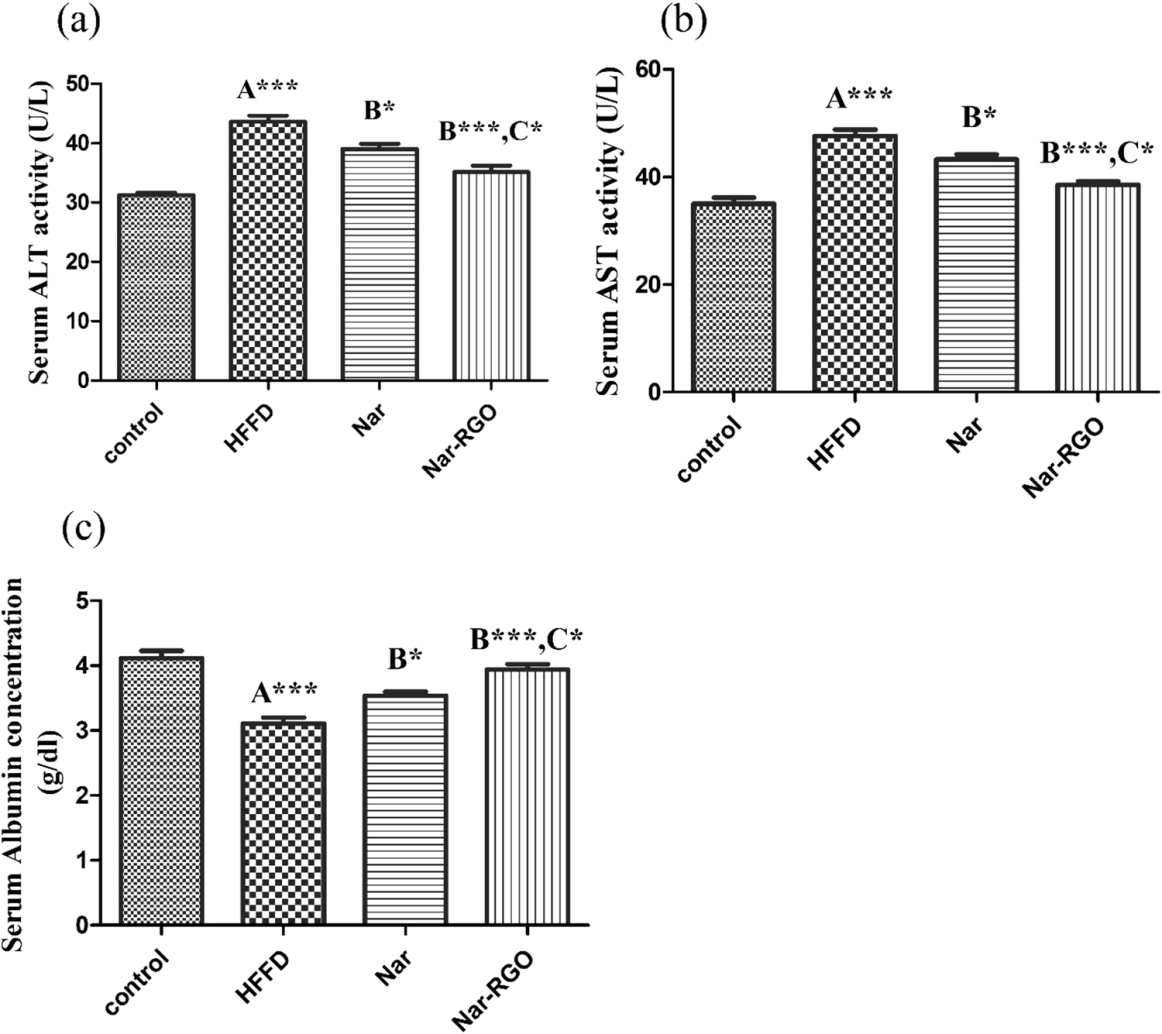
Serum damage markers in rats exposed to HFFD and supplemented with free naringenin and Nar-RGO. **a** Serum ALT activity (U/L), **b** serum AST activity (U/L), and **c** serum albumin concentration (g/dl). Values are presented as mean ± SEM, where letter A means there is significant difference from the control group, letter B means there is significant difference from the HFFD group, and letter C means there is significant difference from the group treated with naringenin 50 mg/kg. ***P > 0.001, **P > 0.01, and *P > 0.05

HFFD feeding resulted in a significant increase in the serum levels of ALT and AST compared to the normal control group (*P*-value > 0.001). The increase in ALT caused by HFFD was significantly ameliorated by the treatment with naringenin (39 ± 0.90, *P* > 0.05) and Nar- RGO (35.12 ± 1.09, *P* > 0.001) compared to the activities in the HFFD group. The effect of Nar-RGO was significantly more potent (*P* > 0.05) than free naringenin. Similarly, the elevated levels of AST were reduced by the concomitant treatment with naringenin (43.25 ± 0.92, *P* > 0.05) and Nar-RGO (38.50 ± 0.68, *P* > 0.001). The effect of Nar-RGO was more significantly potent (*P* > 0.05) as compared to the effect of free naringenin. Conversely, HFFD feeding resulted in a significant decrease in albumin concentration compared to the normal control group (*P* > 0.001). Both forms of naringenin increased albumin concentration (3.53 ± 0.05, *P* > 0.05) and (3.93 ± 0.08, *P* > 0.001), respectively, compared with the HFFD group. The effect of Nar-RGO was significantly greater (*P* > 0.05) compared to that of free naringenin (Fig. 5a–c).

### Effect of naringenin and Nar-RGO on serum lipid profile and adiponectin concentration in rats exposed to HFFD

**Fig. 6:**
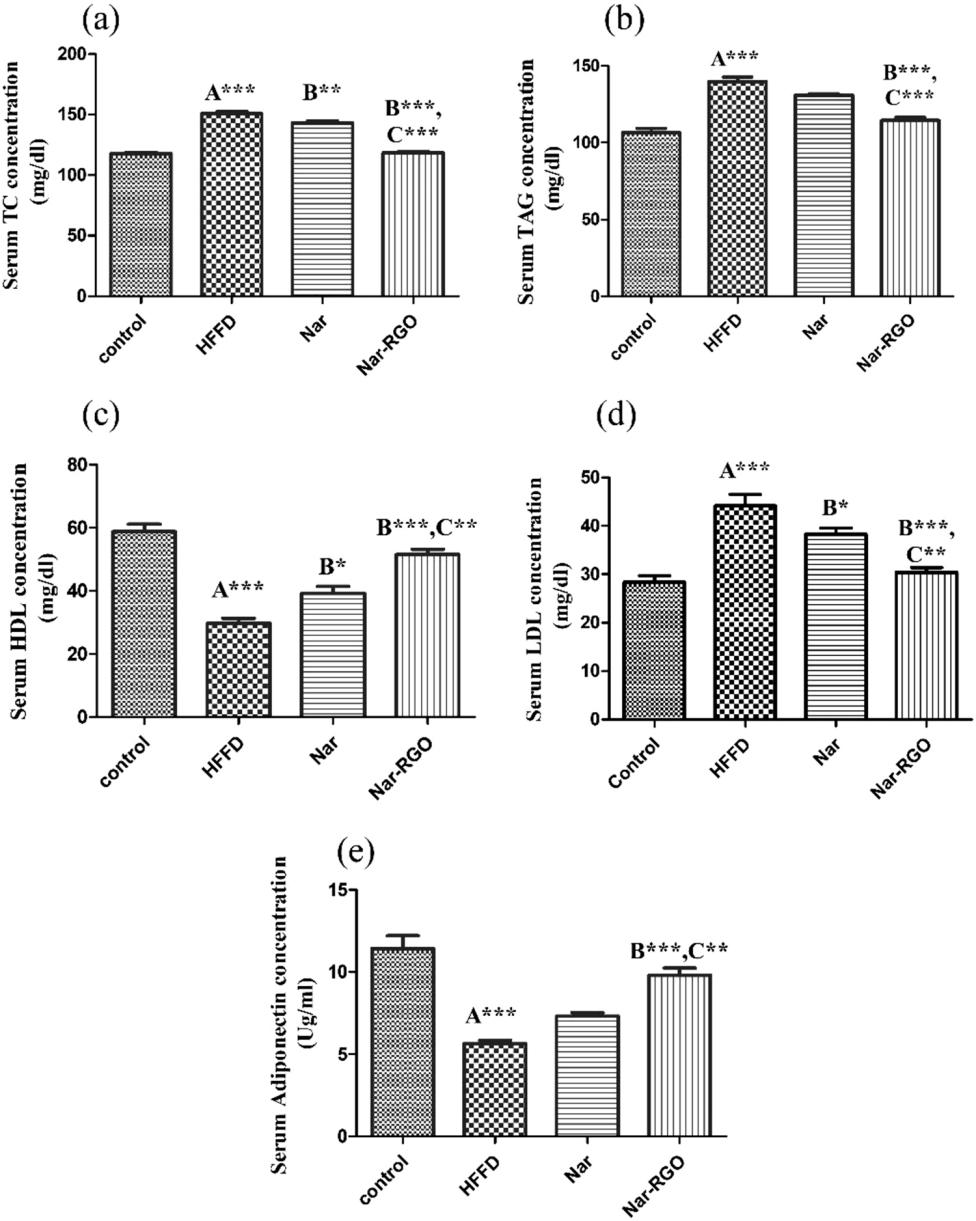
Serum lipid profile and adiponectin concentration in rats exposed to HFFD and supplemented with free naringenin and Nar-RGO. **a** Serum TC concentration (mg/dl), **b** serum TAG concentration (mg/dl), **c** serum LDL-C (mg/dl), **d** serum HDL-C (mg/dl), and serum adiponectin concentration (Ug/ml). Values are presented as mean ± SEM, where the letter A means there is a significant difference from the control group, the letter B means there is a significant difference from the HFFD group, and the letter C means there is a significant difference from the group treated with naringenin 50 mg/kg. ***P > 0.001, **P > 0.01, and *P > 0.05

HFFD feeding resulted in a significant increase in the serum concentrations of TC, TAG, and LDL when compared to the normal control group (*P*-value > 0.001). The increase in TC induced by HFFD was significantly ameliorated by the treatment with naringenin (143 ± 1.53, *P* > 0.01) and Nar-RGO (118.37 ± 1.10, *P* > 0.001) compared to the concentrations in the HFFD group. The effect of Nar-RGO was more significantly potent (*P* > 0.001) compared to the effect of free naringenin. The high concentration of TAG was alleviated by the concomitant treatment with Nar-RGO (114.37 ± 2.04, *P* > 0.001). In contrast, crude naringenin resulted in a non-significant decrease (130.87 ± 1.02, *P* < 0.05). The effect of Nar-RGO was significantly more potent (*P* > 0.001) compared to free naringenin. Conversely, HFFD feeding resulted in a significant decrease in HDL concentration compared to the normal control group (*P* > 0.001). Both forms of naringenin increased HDL concentration (39.25 ± 2.18, *P* > 0.05) and (51.50 ± 1.65, *P* > 0.001), respectively, compared with the HFFD group. The effect of Nar-RGO was significantly more potent (*P* > 0.01) than that of free naringenin. However, there was a substantial decline in adiponectin concentration in HFFD compared to the normal control group (*P*-value > 0.001). This decrease was diminished by consequent treatment with Nar-RGO (9.80 ± 0.43, *P* > 0.001). Meanwhile, treatment with naringenin resulted in a non-significant decrease (*P* > 0.05). The effect of Nar-RGO was more significantly potent (*P* > 0.01) than free naringenin (Fig. 6a–e).

### Effect of naringenin and Nar-RGO on glycemic status in rats exposed to HFFD

**Fig. 7:**
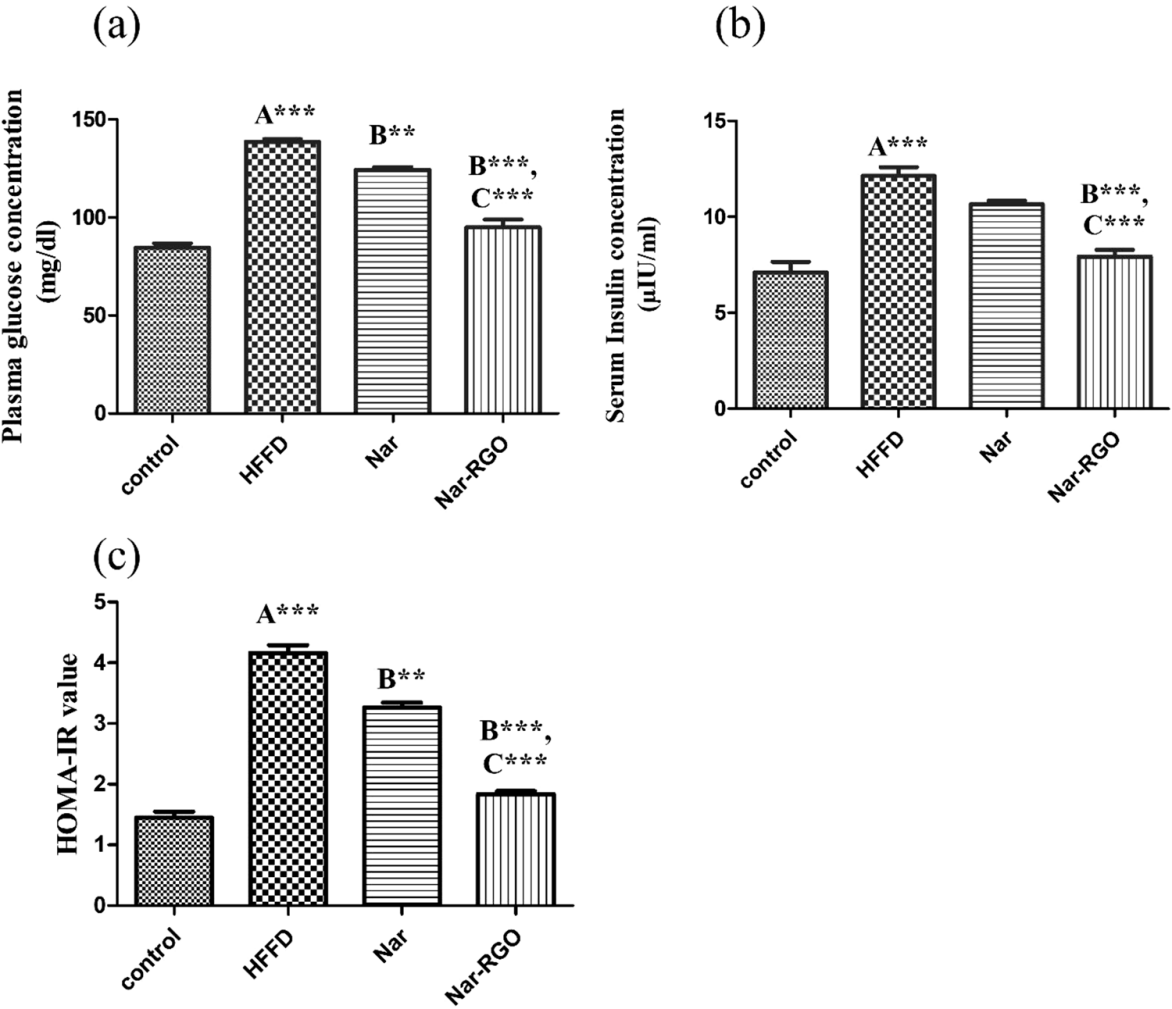
Glycemic status in rats exposed to HFFD and supplemented with naringenin and Nar-RGO. **a** Plasma glucose concentration (mg/dl), **b** serum insulin concentration (µIU/ml), and **c** HOMA-IR value. Values are presented as mean ± SEM, where the letter A means there is a significant difference from the control group, the letter B means there is a significant difference from the HFFD group, and the letter C means there is a significant difference from the group treated with naringenin 50 mg/kg. ***P > 0.001, **P > 0.01, and *P > 0.05

Serum insulin and plasma glucose concentrations were markedly elevated in group II compared to the normal control group (*P* > 0.001). Concomitant treatment with naringenin and Nar-RGO significantly alleviated the hyperglycemia (124.12 ± 1.97, *P* > 0.01) and (94.87 ± 4.08, *P* > 0.001), respectively. Nar-RGO significantly alleviated the hyperinsulinemia (7.92 ± 0.37, *P* > 0.001). Meanwhile, treatment with free naringenin led to a non-significant decrease (10.66 ± 0.18, *P* > 0.05). The Nar-RGO effect was more significantly potent (*P* > 0.001) than free naringenin (Fig. 7a–c).

### Effect of naringenin and Nar-RGO on oxidative stress and antioxidant markers in the liver of rats exposed to HFFD

**Fig. 8:**
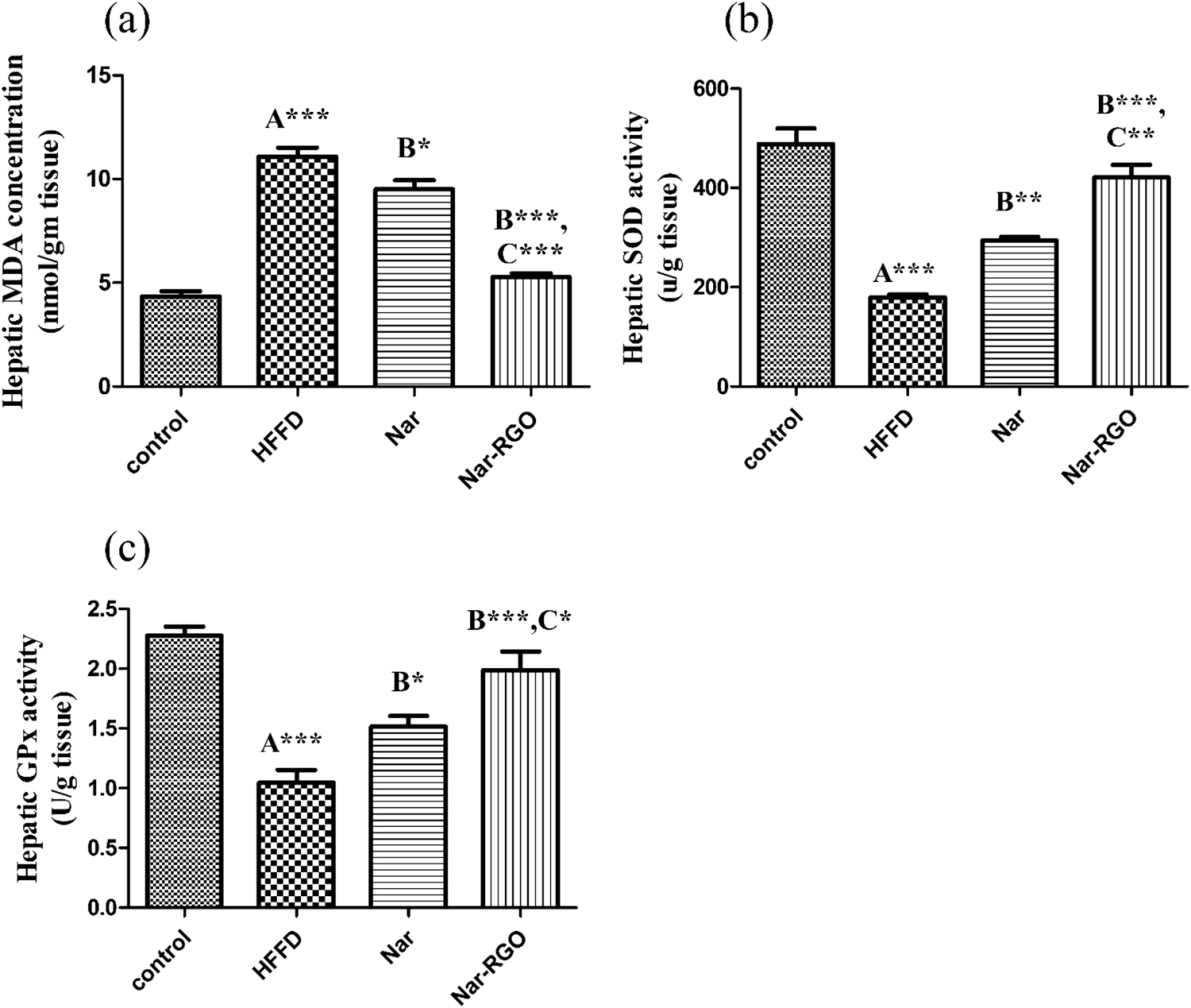
Oxidative stress and antioxidant markers in the liver of rats exposed to HFFD and supplemented with free naringenin and Nar-RGO. **a** Hepatic MDA concentration (nmol/g tissue), **b** hepatic SOD activity (U/g tissue), and **c** hepatic GPx activity (U/g tissue). Values are presented as mean ± SEM, where the letter A means there is a significant difference from the control group, the letter B means there is a significant difference from the HFFD group, and the letter C means there is a significant difference from the group treated with naringenin 50 mg/kg. ***P > 0.001, **P > 0.01, and *P > 0.05

MDA level was markedly increased in the HFFD group compared to the normal control (*P*-value > 0.001). This elevation of MDA was associated with a significant reduction of SOD and GPx activities (*P*-value > 0.001), which reflects the oxidant/antioxidant imbalance in the liver. MDA elevation was alleviated by the concomitant treatment with naringenin (9.50 ± 0.43, *P*-value > 0.05) and Nar-RGO (5.28 ± 0.16, *P*-value > 0.001). The effect of Nar-RGO was more significantly potent (*P* > 0.001) than free naringenin. Additionally, the SOD depletion was attenuated by the administration of naringenin (293.75 ± 7.30, *P*-value > 0.01) and Nar-RGO (421.25 ± 25.03, *P*-value > 0.001). The effect of Nar-RGO was more significantly potent (*P* > 0.01) than free naringenin. Moreover, GPx activities were restored in naringenin (1.51 ± 0.09, *P*-value > 0.05) and Nar-RGO (1.98 ± 0.15, *P*-value > 0.001). The effect of Nar-RGO was more significantly potent (*P* > 0.05) than free naringenin (Fig. 8a–c).

### Effect of naringenin and Nar-RGO on serum levels of proinflammatory cytokines in rats exposed to HFFD

**Fig. 9:**
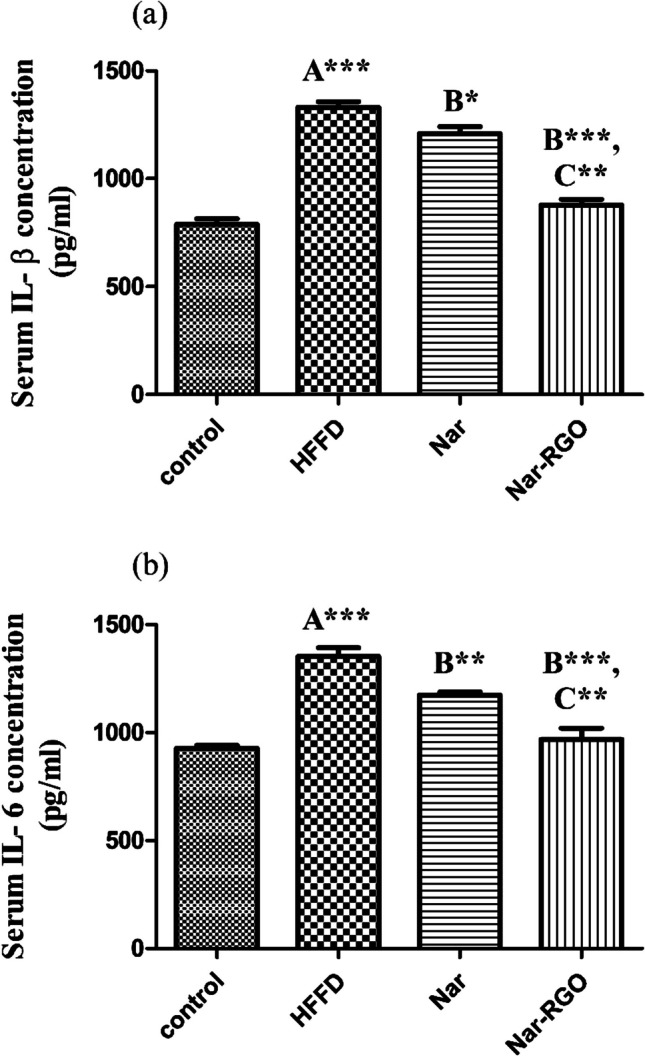
Serum levels of pro-inflammatory cytokines in rats exposed to HFFD and supplemented with free naringenin and Nar-RGO. **a** Serum IL-β (pg/ml) and **b** serum IL-6 (pg/ml). Values are presented as mean ± SEM, where the letter A means there is a significant difference from the control group, the letter B means there is a significant difference from the HFFD group, and the letter C means there is a significant difference from the group treated with naringenin 50 mg/kg. ***P > 0.001, **P > 0.01, and *P > 0.05

HFFD led to a substantial increase in inflammatory cytokine serum levels, including IL-β and IL-6 (*P*-value > 0.001), compared to the control group. Free naringenin at dose 50 mg/kg.bwt led to marked amelioration in the inflammatory response of IL-β (1208.50 ± 32.30, *P*-value > 0.05) and IL-6 (1174.75 ± 12.36, *P*-value > 0.01). However, Nar-RGO resulted in a more significant decline of IL-β (876.25 ± 27.80, *P*-value > 0.001) and IL-6 (967.50 ± 52.74, *P*-value > 0.001). The effect of Nar-RGO was more significantly potent (*P* > 0.01) than free naringenin (Fig. 9a, b).

### Effect of naringenin and Nar-RGO on hepatic gene expressions in rats exposed to HFFD

**Fig. 10:**
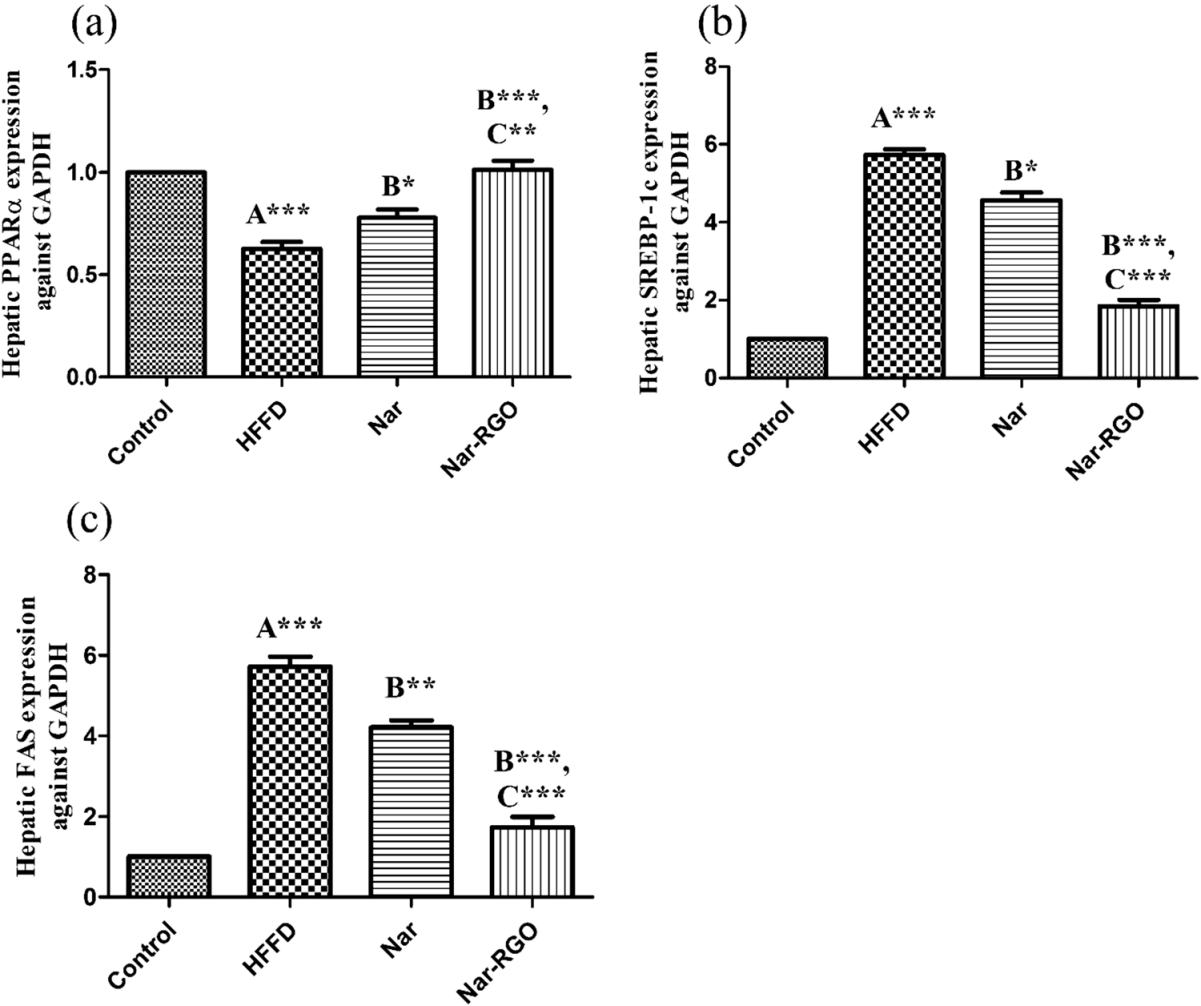
Hepatic gene expressions in rats exposed to HFFD and supplemented with free naringenin and Nar-RGO. **a** PPARα expression against GAPDH, **b** SREBP-1c expression against GAPDH, and **c** FAS expression against GAPDH. Values are presented as mean ± SEM, where the letter A means there is a significant difference from the control group, the letter B means there is a significant difference from the HFFD group, and the letter C means there is a significant difference from the group treated with naringenin 50 mg/kg. ***P > 0.001, **P > 0.01, and *P > 0.05

HFFD resulted in a significant depletion of the m-RNA level of PPAR-α (*P* > 0.001) compared to the control group. The mRNA level of PPAR-α was significantly improved in naringenin (*P<*0.05) and Nar-RGO (*P* > 0.001) when compared to HFFD. The effect of Nar-RGO was more significantly potent (*P* > 0.01) than free naringenin. Conversely, the m-RNA levels of both SREBP-1c and FAS were significantly aggravated in HFFD (*P* > 0.001) compared to HFFD. Cotreatment with free naringenin significantly decreased the elevation of SREBP-1c and FAS, with (*P* > 0.05) and (P > 0.01), respectively. Nar-RGO has a notable superior effect over naringenin in the mitigation of both SREBP-1c and FAS (*P* > 0.001) (Fig. 10a–c).

### H&E histopathological examination of hepatic tissues in rats exposed to HFFD and supplemented with free naringenin and Nar-RGO

**Fig. 11:**
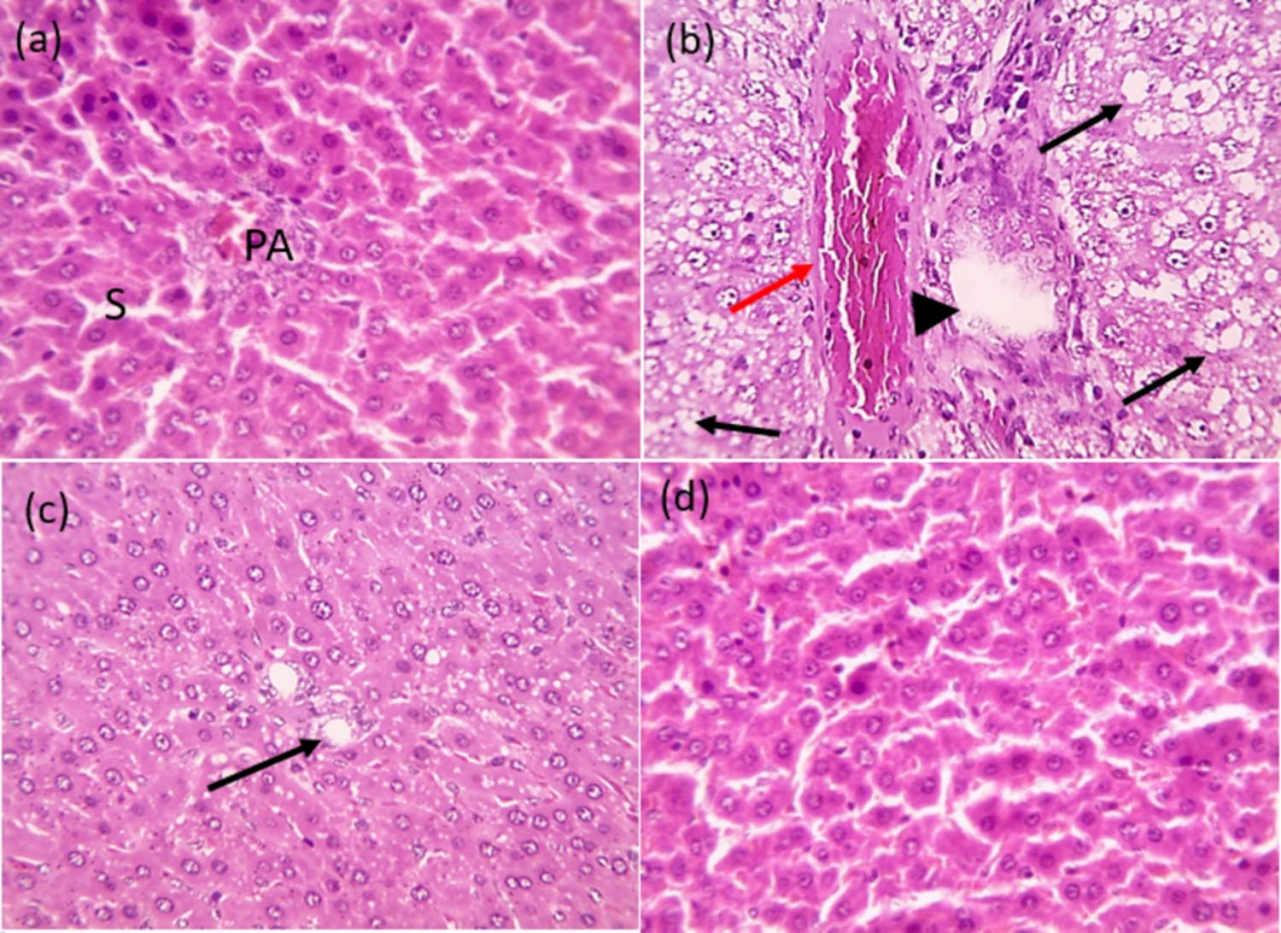
H&E histopathological examination of hepatic tissues in rats exposed to HFFD and supplemented with free naringenin and Nar-RGO. Microscopic pictures of H&E-stained hepatic sections showing a normal arrangement of hepatic cords with normal central veins, portal areas (PA), and sinusoids (s) in the control group (**a**) and the group received Nar-RGO (**d**). Hepatic sections from the group that received HFFD (**b**) showing disrupted hepatic parenchyma, marked cell swelling (macrovesicular steatosis) (black arrows) and hydropic degeneration of hepatocytes in the periportal zone, congested blood vessels (red arrows), mildly dilated bile ductule (arrowhead). Meanwhile, hepatic sections from naringenin received group (**c**) shows mild cytoplasmic vacuolization (macrovesicular steatosis) of a few hepatocytes in the periportal zone (black arrows). X: 400 bar 50

Microscopic images of H&E stained hepatic sections revealed the normal arrangement of hepatic cords with normal central veins, portal areas (PA), and sinusoids (s) in both the control group and the group that received Nar-RGO, as depicted in Fig. 11a, d. In Fig. 11b, the hepatic sections from the group that received HFFD exhibit disrupted hepatic parenchyma, significant cell swelling (macrovesicular steatosis) indicated by black arrows, and hydropic degeneration of hepatocytes in the periportal zone. Additionally, congested blood vessels are observed (red arrows) along with a mildly dilated bile ductule (arrowhead). Meanwhile, the hepatic sections from the group that received naringenin showed mild cytoplasmic vacuolization (macrovesicular steatosis) of a few hepatocytes in the periportal zone (black arrows), as shown in Fig. 11c.

### Oil red O histopathological examination of hepatic tissues in rats exposed to HFFD and supplemented with free naringenin and Nar-RGO

**Fig. 12:**
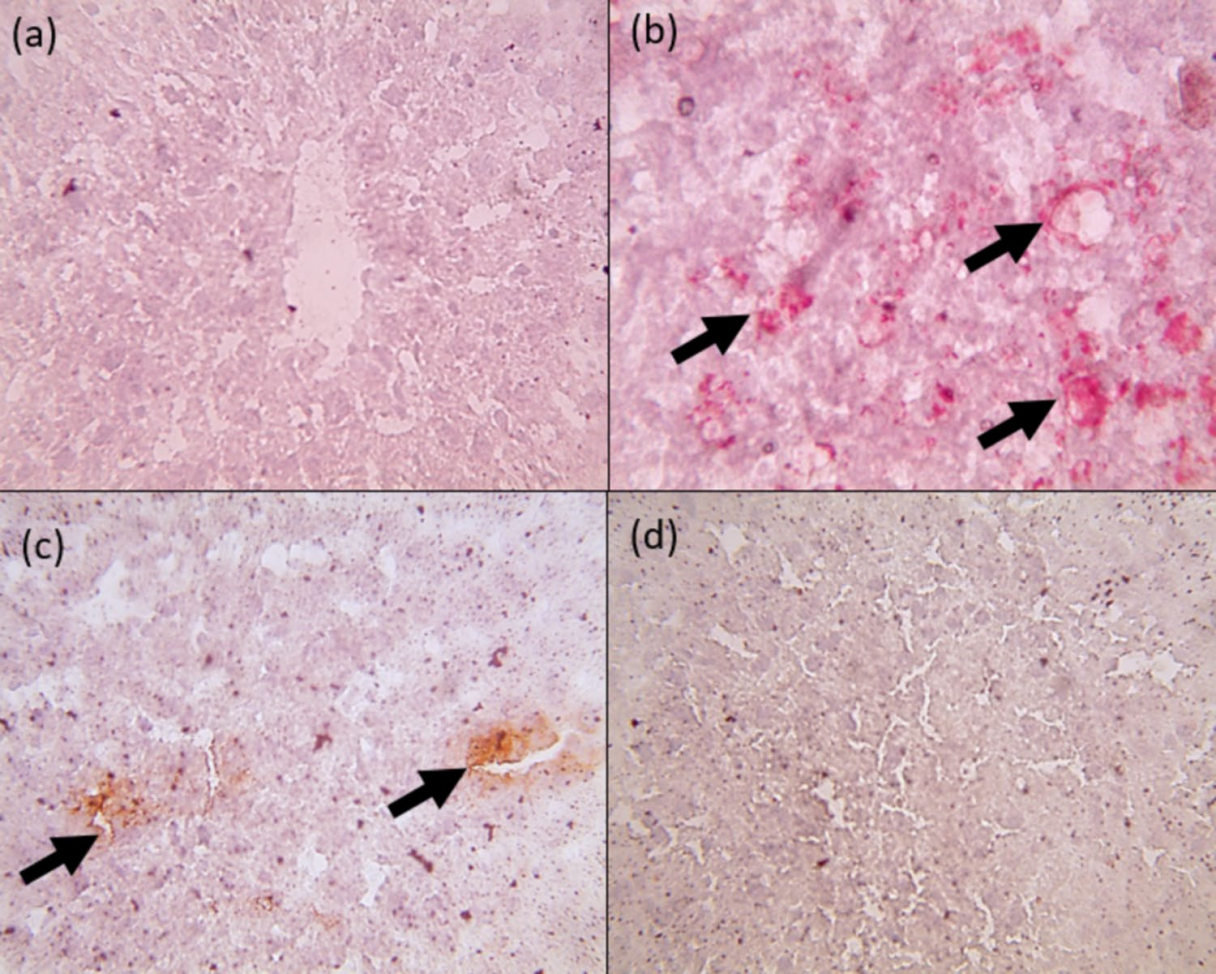
Oil red O histopathological examination of hepatic tissues in rats exposed to HFFD and supplemented with free naringenin and Nar-RGO. ORO-stained hepatic sections showed no fat deposition in the control group (**a**), and the group received Nar- RGO (**d**). Hepatic sections from the group that received HFFD (**b**) showed marked deposition of red-stained fat globules in hepatocytes. Meanwhile, some ORO-stained hepatic sections from the naringenin received group showing mild fat deposition (**c**)

Hepatic sections stained with oil red O demonstrate the absence of fat accumulation in the control group and the group treated with Nar-RGO, as shown in Fig. 12a, d. Hepatic sections from the group that received HFFD demonstrated marked deposition of red-stained fat globules in hepatocytes, as shown in Fig. 12b. In addition, some oil red O stained hepatic sections from the group treated with naringenin exhibited mild fat deposition, as depicted in Fig. 12c.

## Discussion

In recent years, long-term medication treatment regimens for NAFLD have been associated with drug resistance and adverse effects in the majority of patients. As a result, there has been a shift toward the utilization of adjuvant medical treatments derived from natural products (Y.-J. Zhou et al. 2021). Numerous studies have confirmed the hepatoprotective, hypoglycemic, antioxidant, and anti-inflammatory roles of natural bioactive flavonoid naringenin (Hassan et al. 2021; Hernández-Aquino and Muriel 2018; Yadav et al. 2020). Flavonoid-2D hybrid nanocomposite has attracted much appreciation in recent years because of their combinational effects (Shanmuganathan et al. 2020). RGO is a two-dimensional material well known for its biocompatibility and suitability for drug delivery applications. Its high surface-to-volume ratio enables more effective interactions with drugs (Shirsat and Hianik 2023). Additionally, it is recognized for its antioxidant and anti-inflammatory properties (Di Mauro et al. 2023). This study utilized natural flavone naringenin for the reduction of graphene oxide and emphasized the key benefits of employing Nar-RGO in comparison to free naringenin for mitigating HFFD-induced NAFLD. To our knowledge, this is the first study that suggested the beneficial effects of Nar-RGO on hepatic steatosis.

Initially, Nar-RGO was characterized using SEM and TEM analyses, which demonstrated the effective incorporation of naringenin within the RGO structure. Furthermore, the stability of the Nar-RGO was assessed by measuring the zeta potential. The formulation exhibits a negative zeta potential (− 50.8 mv). Graphene oxide typically exhibits a negative zeta potential (− 31 to – 33 mV) due to the presence of oxygen-containing functional groups on its surface, such as carboxyl, hydroxyl, and epoxy group (Baskoro et al. 2018). The interaction between graphene oxide and naringenin enhances the negative charge which increase zeta potential to be more negative. This leads to the stable dispersion of RGO in an aqueous solution by the electrostatic repulsion interaction with water molecules (Sadhukhan et al. 2016). These results are in agreement with findings from Murugan et al. (2023), who investigated the targeted brain delivery of Nar-RGO (Murugan et al. 2023).

The high-fat fructose model of non-alcoholic fatty liver disease (NAFLD) is frequently utilized in research due to its reliability and reproducibility, as well as its association with dyslipidemia, inflammation, and oxidative stress (Buniam et al. 2023; Rosita et al. 2021; Zhou et al. 2022). The present study found that HFFD resulted in liver damage, oxidative stress, and mitochondrial dysfunction due to excessive accumulation of fat in the liver. This was confirmed by elevated TAG levels and the presence of fatty droplets detected by H&E and oil red O staining. When the liver is damaged, the enzymes ALT and AST are released from the hepatocytes into the bloodstream. This leads to an increase in their levels in the serum, indicating that the hepatocytes have been harmed. The damage includes dysfunction of the mitochondria and necrosis of the hepatocytes. As a result, the permeability of the cell membrane increases, causing the leakage of these intracellular transaminases into the bloodstream (Attia et al. 2022). These findings are in accordance with those of Amir Siddiqui et al. (2022) and Yuvaraj et al. (2021). Crude naringenin at (50 mg/kg/per day) repressed serum levels of liver enzymes, though it did not inhibit hepatic lipid accumulation. Furthermore, Nar-RGO significantly reduced serum liver enzymes and alleviated hepatic lipid accumulation. The TAG reduction capacity of naringenin could be attributed to the ability of naringenin to stimulate TAG oxidation and inhibit TAG synthesis (Cho et al. 2011). The significant enhancement of the Nar-RGO in TAG reduction compared with the crude naringenin was attributed to the large surface area of RGO, which provides more sites for naringenin molecules to adhere to and interact with, producing more effective action.

Hepatic lipid homeostasis imbalance, particularly DNL aggravation, leads to the excessive accumulation of lipids in hepatocytes. This is believed to be the primary cause of the development of NAFLD. DNL plays a role in the development of NAFLD by affecting various metabolic pathways, such as glycolysis, pyruvate oxidation, synthesis of saturated fatty acids, and formation of triglycerides (Chen 2020). Through a gene expression analysis and histopathological examination, it can be inferred that the HFFD-treated group experienced increased lipid accumulation due to heightened SREBP-1c and FAS activities. However, these levels were reduced after naringenin therapy, particularly Nar-RGO. The mechanisms underlying the hypolipidemic effects of naringenin and Nar-RGO may involve downregulating the activity of lipogenic enzymes such as SREBP-1c, a key transcription factor that regulates the expression of DNL rate-limiting enzymes like Fas and Acc1. These enzymes contribute to the pathophysiology of NAFLD and hepatic steatosis (Zhou et al. 2020). In a recent study, it was found that the presence of graphene as the core structure significantly contributes to lipolytic activity by binding to adipocyte integrin β1 (de Frutos et al. 2023). Similarly, in ethanol-treated HepG2 cells, it was observed that graphene oxide enhances lipolytic activity through the downregulation of SREBP-1c (Aghara et al. 2024).

Oxidative stress is a significant contributor to the pathophysiology of NAFLD (Abd El-Haleim et al. 2016). The elevated hepatic MDA levels seen in this study suggest the presence of oxidative stress. This is supported by the concurrent reduction in the activities of GPx and SOD, which are crucial cellular antioxidants that counteract redox imbalance and protect cells against oxidative damage. SOD is a critical enzyme that converts superoxide radicals to molecular oxygen and hydrogen peroxide. GPx is an enzyme that converts lipid hydroperoxides to water (Kandemir et al. 2017). Our study revealed that Nar-RGO offers an effective defense against HFFD-induced lipid peroxidation compared to crude naringenin. This finding can be ascribed to the substantial surface area, which may improve the interaction between naringenin and reactive oxygen species (ROS). This interaction can increase the efficiency of naringenin in scavenging ROS, thereby improving its antioxidant activity. Additionally, reduced graphene oxide shows considerable radical scavenging activity, which results in a notable decrease in ROS production, according to the study by Qiu et al. (2014).

Adiponectin, primarily released by adipose tissue, plays a vital role in the onset and progression of fatty liver disease. It exerts anti-inflammatory effects on the liver by inhibiting the production of proinflammatory cytokines such as IL-1β, IL-6, and TNF-α (Akbari et al. 2021). Therefore, serum levels of proinflammatory cytokines, such as IL-6 and IL-1β, were further exacerbated in HFFD-treated rats due to the formation of ROS triggered by liver injury, development of simple steatosis to steatohepatitis, and low levels of adiponectin. Naringenin, particularly Nar-RGO, significantly reduced the inflammatory response and adiponectin reduction caused by liver injury. This result can be attributed to the sustained supply of naringenin encapsulated by reduced graphene oxide. This result can be attributed to the reduction of oxidative stress, which is a key contributor to inflammatory responses.

In the HFFD group, hypertriglyceridemia is accompanied by hyperglycemia and hyperinsulinemia, as well as insulin resistance. These findings could be attributed to the lipotoxicity hypothesis, which posits that high fat and carbohydrate load may alter the lipid and protein composition of the membrane system. Alternation in membrane integrity might impact insulin receptors and glucose transporter protein. As a result, the muscle’s glucose uptake decreased, which raised plasma glucose levels more dramatically (Brøns and Grunnet 2017). Therefore, adiponectin improves hepatic and peripheral insulin resistance (Zhang et al. 2019). Both serum cholesterol and triglyceride levels were found to be significantly higher in HFFD-induced diabetic rats in comparison with the respective control group. Improper glucose functioning and metabolism cause hypertriglyceridemia and hypercholesterolemia (Sen et al. 2011). These levels were normalized by Nar-RGO. However, the levels remained significantly higher in the group that received crude naringenin. The decrease in elevated blood glucose and downregulation of tri acyl glycerol and cholesterol concentrations could be due to the stimulatory activity of naringenin by pancreatic β cell regeneration (Maity and Chakraborti 2020). In summary, the unique properties of reduced graphene oxide, particularly in combination with naringenin, highlight its potential against HFFD-induced NAFLD. A notable limitation of our study is the lack of sampling from different organs, especially the kidneys, which is essential for fully assessing the safety of Nar-RGO for future applications.

## Conclusion

Though challenges in scalability, cost, and safety persist, reduced graphene oxide possesses notable features, including high surface area, enhanced electrical conductivity, mechanical strength, and chemical versatility. Conventional reduction methods used for RGO synthesis may show toxicity risks. Therefore, in our study, we utilize naringenin as a safe reducing agent to synthesize RGO. Additionally, we assess the benefits of naringenin-reduced graphene oxide by evaluating its effects on non-alcoholic fatty liver disease. This study suggests that the utilization of Nar-RGO resulted in a significant improvement in hepatic steatosis, insulin resistance, oxidative stress, and signs of inflammation in HFFD-induced NASH compared to crude naringenin.

## Data Availability

No datasets were generated or analysed during the current study.
